# Global research hotspots and trends in the field of spine surgery during the COVID-19 pandemic: A bibliometric and visual analysis

**DOI:** 10.3389/fsurg.2022.976546

**Published:** 2022-09-09

**Authors:** Guang-Xun Lin, Vit Kotheeranurak, Chien-Min Chen, Bao-Shan Hu, Gang Rui

**Affiliations:** ^1^ Department of Orthopedics, The First Affiliated Hospital of Xiamen University, School of Medicine, Xiamen University, Xiamen, China; ^2^ The Third Clinical Medical College, Fujian Medical University, Fuzhou, China; ^3^ Department of Orthopaedics, Faculty of Medicine, Chulalongkorn University, and King Chulalongkorn Memorial Hospital, Bangkok, Thailand; ^4^ Center of Excellence in Biomechanics and Innovative Spine Surgery, Chulalongkorn University, Bangkok, Thailand; ^5^ Division of Neurosurgery, Department of Surgery, Changhua Christian Hospital, Changhua, Taiwan; ^6^ Department of Leisure Industry Management, National Chin-Yi University of Technology, Taichung, Taiwan; ^7^ School of Medicine, Kaohsiung Medical University, Kaohsiung, Taiwan

**Keywords:** COVID-19, spine surgery, bibliometric analysis, research trends, SARS-CoV-2

## Abstract

**Background:**

The Coronavirus disease-2019 (COVID-19) significantly affected the healthcare and research systems, including spine surgery, throughout the world. A bibliometric analysis allows graphical visualization of the development of an academic field and its frontiers. Since research concerning spine surgery during the COVID-19 pandemic is being constantly upgraded, we conducted a bibliometric analysis of this literature to investigate the current status, research hotspots, and trends in this field.

**Methods:**

We searched the Web of Science database for literature published (from December 1, 2019, to March 24, 2022) using the terms “COVID-19” OR “2019-nCoV” OR “SARS-CoV-2” AND “spine surgery” OR “spinal surgery” OR “discectomy” OR “decompression” OR “laminectomy” OR “interbody fusion” OR “pedicle screws.” Detailed bibliometric and visual analysis of the number of publications, geographical distribution, institutions, journals, authors, and keywords was done using CiteSpace, VOSviewer, and R-Bibliometrix.

**Results:**

Of the initially screened 173 articles, we included 84 relevant articles—62 original articles, 10 editorial materials, 8 reviews, and 4 others. The United States, China, Egypt, and Argentina were most actively publishing in the field of spine surgery and COVID-19. The AOSpine International community contributed 7 articles (24 citations). The Hospital for Special Surgery (13.1%) and Johns Hopkins University (13.1%) were institutions with the most publications. Using the Law of Bradford, we found that *World Neurosurgery, Global Spine Journal, and European Spine Journal* are the core journals in this field, with *P*. K. Louie being the most influential author. “Elective surgery,” “intensive care,” “telehealth,” “patient satisfaction,” and “follow-up” had the strongest citation bursts.

**Conclusions:**

During the COVID-19 pandemic, spine surgeons were more concerned with surgical timing, care, treatment, and patient’s quality of life. Accordingly, research hotspots in spine surgery during the pandemic shifted from “early healthcare” to “virus management” and “experience and education.”

## Introduction

The recent coronavirus disease 2019 (COVID-19) pandemic was caused by a global rampant spread of the severe acute respiratory syndrome coronavirus 2 (SARS-CoV-2), sending the health systems into a state of emergency throughout the world (1–3). Patients with COVID-19 infection present with varying symptoms, ranging from sore throat, sneezing and runny nose, fever, cough, shortness of breath, fatigue, muscle pain, body aches, headache, nausea, and diarrhea to severe infection resulting in death (4–6). With subsequent mutations and infection with the novel Omicron variant, the normalization of outbreak prevention methods and controls has entered a new phase.

Spine surgery has been one of the major surgical specialties facing enormous demands from the community throughout the epidemic due to its relevance in a wide spectrum of diseases, including both emergency care, such as trauma, and elective surgeries for infection, intractable pain, and severe neurological symptoms of spinal diseases (7–9). Therefore, the need to balance the social pressures for competent medical attention while dispensing safe and effective surgical care during the current phase of implementing cautious infection prevention and control strategies has placed higher demands on the existing treatment processes and protective measures followed in spine surgery (9–11).

Driven by these issues, studies related to the field of spine surgery during the pandemic have been published and the topic has received increasing attention from researchers. Bibliometric analysis can provide researchers with a lot of meaningful information. Bibliometrics is a computer-based statistical tool that enables investigators to quantitatively analyze the extent of research publications on a specific topic (12). It can also be used to assess the quality of a study, discern the major research themes in a particular area, and predict the direction of future research (13). The major components of bibliometrics include total documentation, collaboration analysis, influence analysis, keyword analysis, and citation relationship network (14). The Web of Science (WoS) database (Thomson Reuters, New York, NY) allows access to almost all key scientific publications along with built-in analysis tools, making it a database widely used for bibliometric analysis.

Since the COVID-19 pandemic is still evolving and not completely under control, continuing research is required to update the current practices in light of this disease, Therefore, we found it pertinent to conduct a comprehensive and meticulous bibliometric analysis of research in the field of spine surgery during the COVID-19 pandemic, and determine the research hotspots and trends from the perspective of spine surgeons. We believe this information may be useful and valuable for spinal surgeons in determining the future course of research.

## Materials and methods

We searched the WoS Core Collection using the following keywords to include studies published from December 1, 2019, to March 24, 2022: “COVID-19” OR “2019-nCoV” OR “SARS-CoV-2” AND “spine surgery” OR “spinal surgery” OR “spinal cord injury” OR “spinal fracture” OR “discectomy” OR “decompression” OR “laminectomy” OR “interbody fusion” OR “pedicle screws.” The articles so obtained were chosen and evaluated separately by two scholars; any disagreements regarding inclusion were resolved through discussion.

We used CiteSpace (Chaomei Chen, Drexel University, USA), VOSviewer (version 1.6.18), and the R-bibliometrix (v4.1.3, R Foundation, Vienna, Austria), for bibliometric analysis and its visualization using network maps, which allowed for better comprehension and interpretation of subjects. We analyzed the document type, language, journal, region, institution, author, and keywords for each publication. VOSviewer was used to visualize the region, author and institutional co-authorship, source co-citation, and keyword co-occurrence, and to make network maps. Different nodes in the network visualization map provided by VOSviewer indicated various factors, such as nationality, number of journals, and keywords. The size of the nodes in a visual network diagram represents the degree of co-occurrence or citation frequency, i.e., the number of publications or occurrences was proportional to the size of the map’s nodes. The node connection represents a relationship between author cooperation, co-occurrence, or co-citation. The lines represent the connections between the nodes, and their colors represent the year of publication. The thickness of the linkages and the distance between nodes reflects how closely different nations, institutions, and writers collaborate. We used the Bibliometrix package to compute descriptive statistics and perform network analysis of the documentary data and organize and visualize it accordingly. Additionally, CiteSpace allowed performing burst detection on keywords.

This study is essentially descriptive research without statistical analysis; the quantity and ratio (proportion) of each indicator indicate the distribution and evolving trends over different years, nationalities, institutions, journals, and authors.

## Results

### Publication output

Initially, 173 publications published between December 1, 2019 and March 24, 2022, were retrieved from the WoS database and carefully screened by both scholars independently. After excluding the non-relevant papers, 84 publications were identified on the combined subjects of spine surgery and COVID-19, which included 62 (73.8%) original articles, 10 (11.9%) editorial materials, 8 (9.5%) review articles, 3 (3.6%) letters, and 1 (1.2%) conference abstract. All publications were in English. Of these, 35 papers were published in 2020, 43 in 2021, and 6 papers in 2022 (until March 24, 2022).

### Number of publications for different countries/regions

The included studies were conducted across 30 countries of the world. The top 10 publishing in the field of spine surgery and COVID-19 were the United States (*n* = 41, 48.8%), China (*n* = 11, 13.1%), Egypt (*n* = 10, 11.9%), Argentina (*n* = 8, 9.5%), Switzerland (*n* = 8, 9.5%), Italy (*n* = 8, 9.5%), Finland (*n* = 7, 8.3%), India (*n* = 5, 5.9%), Germany (*n* = 4, 4.8%), Canada (*n* = 4, 4.8%), and Singapore (*n* = 4, 4.8%).

**Figure 1:**
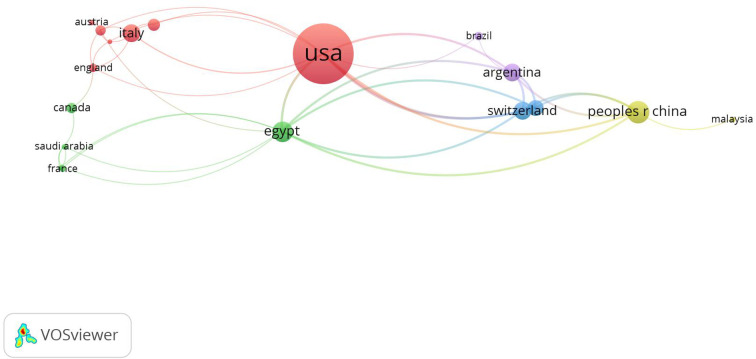
International collaboration analysis among different countries/regions.

A map of the international cooperation between relevant countries/regions shows that the United States co-operates more closely with the top 5 countries in terms of publications (Figure 1).

### Analysis of institutions

**Figure 2:**
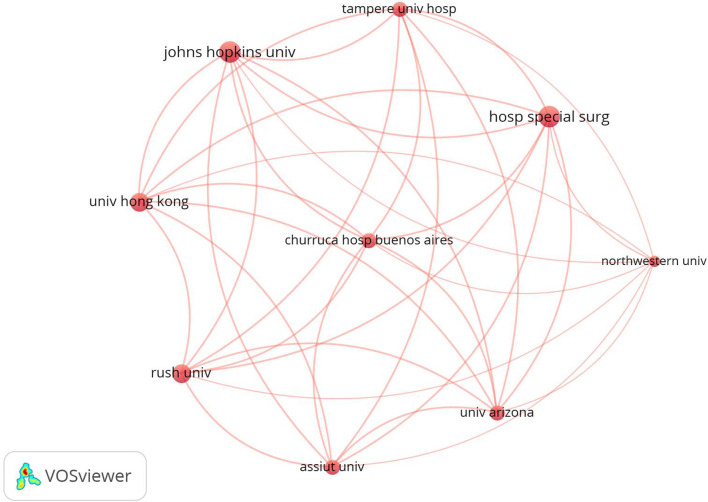
Co-operation network of productive institutions.

An institution-wise analysis revealed that an estimated 215 institutions contributed to research in this field. Table 1 presents a list of different institutions ranked according to the number of documents in the field of spine surgery and COVID-19. Of the 84 publications included in this study, the maximum number of articles were published by the Hospital for Special Surgery and Johns Hopkins University (*n* = 11 articles, 13.1% each), followed by Rush University and the University of Hong Kong (*n* = 9 articles, 10.7% each). VOSviewer generated a visual map of inter-institutional collaboration (Figure 2); the top 10 institutions with the most published articles collaborated closely with each other and were mostly concentrated in the United States.

**Table 1:** Top 10 list of number of articles by institution.

Rank	Institution	Documents
1	Hospital for Special Surgery	11
2	Johns Hopkins University	11
3	Rush University	9
4	University of Hong Kong	9
5	University of Arizona	7
6	Assiut University	7
7	Hospital Churruca	7
8	Tampere University	7
9	Northwestern University	5
10	Harvard University	3

In addition, the AOSpine International community contributed seven articles with 24 citations.

### Analysis of journals

**Figure 3:**
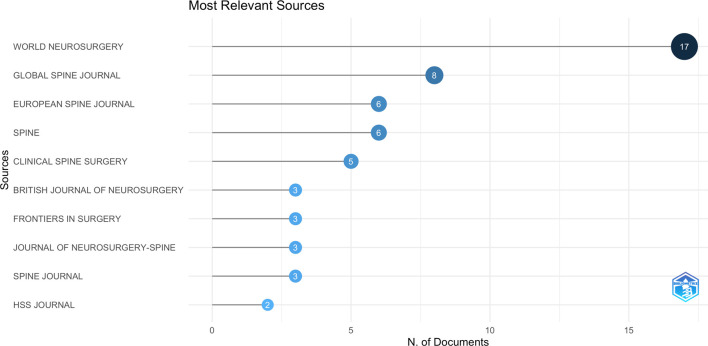
Top 10 journals with the highest number of publications in the field of spine surgery and COVID-19 (generated by Bibliometrix).

**Figure 4:**
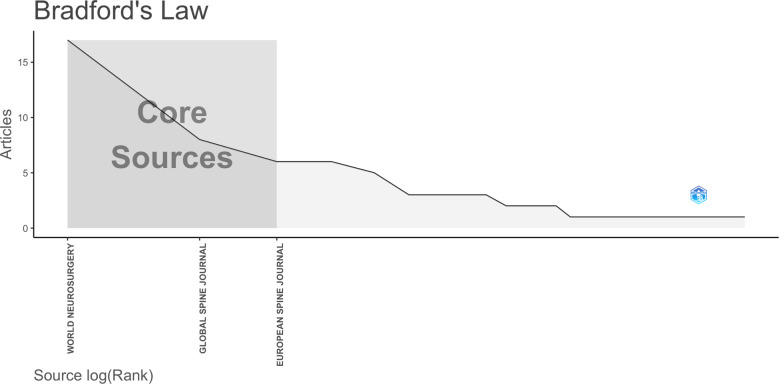
Bradford’s Low map. The journal located in Core Sources is this journal published a larger number of articles on spine surgery and COVID-19, indicating it as a core journal in this field (generated by Bibliometrix).

Figure 3 presents a list of the top 10 journals with the highest number of published articles in the field of spine surgery and COVID-19. The maximum number of articles were published in *World Neurosurgery* (*n* = 17, 20.2%), followed by the *Global Spine Journal* (*n* = 8, 9.5%), *European Spine Journal* (*n* = 6, 7.1%), *Spine* (*n* = 6, 7.1%), and *Clinical Spine Surgery* (*n* = 5, 6.0%). A descending graph based on the Law of Bradford in bibliometric analysis revealed that *World Neurosurgery*, *Global Spine Journal*, and *European Spine Journal* were the core journals in the field of spine surgery and COVID-19 (Figure 4). Articles published in these essential journals received more attention, and therefore, were cited more frequently than articles in other journals.

### Analysis of authors

**Figure 5:**
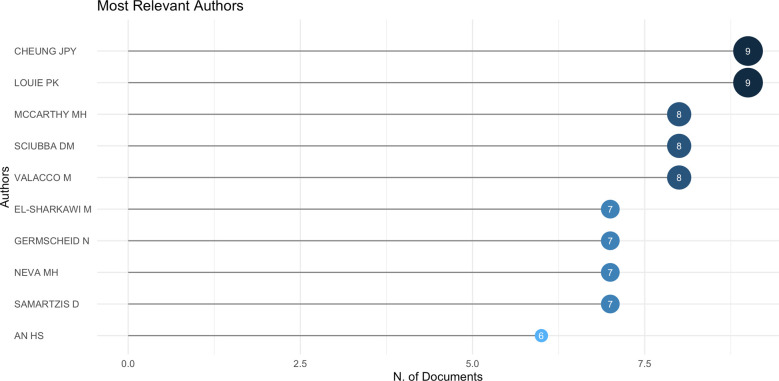
Top 10 authors with the highest number of publications in the field of spine surgery and COVID-19 (generated by Bibliometrix).

Figure 5 presents the top 10 authors with the most publications in this field. During the COVID-19 pandemic, *P*. K. Louie and. *P*. Y. Cheung were the most productive authors in the fields of spine surgery and COVID-19 (*n* = 9, 10.7%), followed by M. H. McCarthy (*n* = 8, 9.5%), M. Valacco (*n* = 8, 9.5%), and D. M. Sciubba (*n* = 7, 8.3%). Interestingly, most of these authors are AOSpine members and have co-authored many articles.

**Figure 6:**
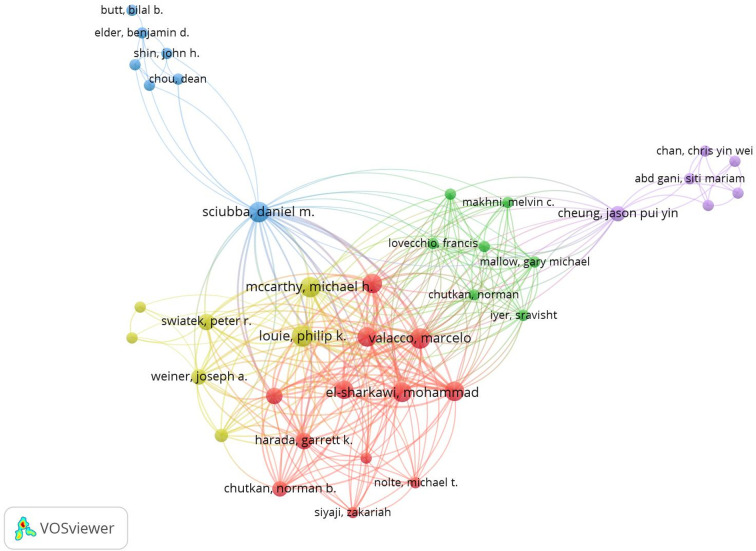
Overlay visualization map of author co-authorship analysis.

**Figure 7:**
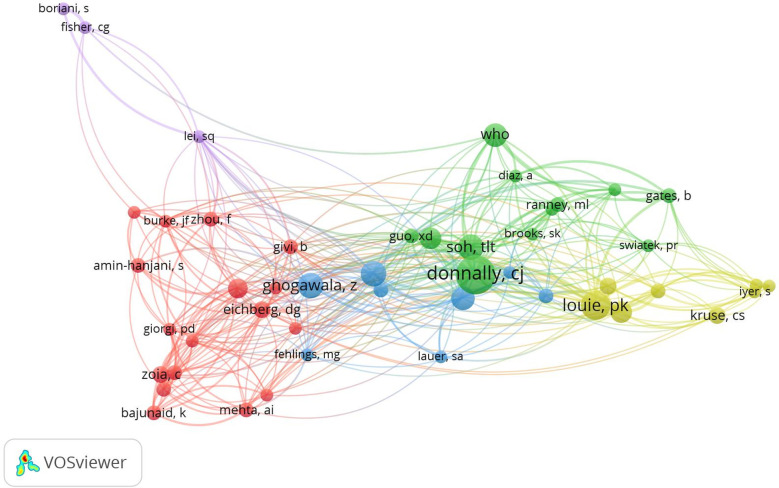
Visualization map of author co-citation analysis.

Author co-citation analysis is commonly used to identify the important authors in a field’s co-citation network. From the visual map of the author–co-authorship analysis (Figure 6), we found that these authors created several research clusters—different colors represent different theme clusters. Each cluster is radiated by two or three core authors; some authors were connected between different clusters, indicating a better collaboration in this field. In general, frequently cited authors have a greater influence in the field. The greater the node, the more references to the author (Figure 7). The top two most co-cited authors in the field of spine surgery and COVID-19 were *P*. K. Louie and C. J. Donnally.

### Analysis of citations

The maximum and the minimum number of citations of the included articles were 57 and 14, respectively. Of the 10 most cited articles (Table 2), 6 were from the United States, and the remaining 4 were one article from Singapore, India, Australia, and Italy. Four of these top 10 articles were published in the *World Neurosurgery* journal.

**Table 2:** Top 10 most cited articles in the field of spine surgery during the COVID-19 pandemic.

Rank	Title	Authors	Institution of the corresponding author	Country of the corresponding author	Journal	Year	Citation
1	SARS-CoV-2 Impact on Elective Orthopaedic Surgery Implications for Post-Pandemic Recovery	A. Jain et al.	Johns Hopkins University	USA	Journal of Bone and Joint Surgery-American Volume	2020	57
2	Triaging Spine Surgery in the COVID-19 Era	C. J. Donnally et al.	Thomas Jefferson University	USA	Clinical Spine Surgery	2020	33
3	Telemedicine in Neurosurgery: Lessons Learned and Transformation of Care During the COVID-19 Pandemic	N. Mouchtouris et al.	Thomas Jefferson University	USA	World Neurosurgery	2020	30
4	COVID-19 and spinal surgery	Z. Ghogawala et al.	Lahey Hospital and Medical Center	USA	Journal of Neurosurgery-Spine	2020	24
5	Virtual Spine: A Novel, International Teleconferencing Program Developed to Increase the Accessibility of Spine Education During the COVID-19 Pandemic	J. J. Rasouli et al.	Cleveland Clinic	USA	World Neurosurgery	2020	18
6	Collateral damage caused by COVID-19: Change in volume and spectrum of neurosurgery patients	N. Goyal et al.	All India Institute of Medical Sciences	India	Journal of Clinical Neuroscience	2020	16
7	An Australian Response to the COVID-19 Pandemic and Its Implications on the Practice of Neurosurgery	J. Antony et al.	Gold Coast University Hospital, The University of Queensland	Australia	World Neurosurgery	2020	16
8	Spine Surgery and COVID-19 Challenges and Strategies from the Front Lines	T. L. T. Soh et al.	Tan Tock Seng Hospital	Singapore	Journal of Bone and Joint Surgery-American Volume	2020	16
9	Sheltered Neurosurgery During COVID-19: The Emory Experience	H. Saad et al.	Emory University	USA	World Neurosurgery	2020	15
10	The management of emergency spinal surgery during the COVID-19 pandemic in Italy	*P*. D. Giorgi et al.	A.S.S.T. Grande Ospedale Metropolitano Niguarda	Italy	Bone / Joint Journal	2020	14

### Analysis of keywords and research hotspots

**Figure 8:**
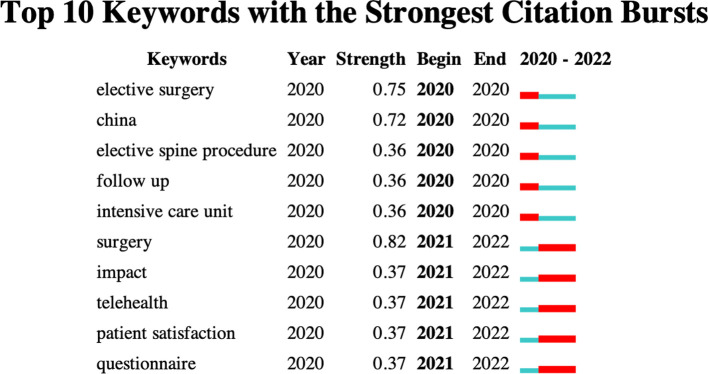
Top 10 keywords with the strongest citation bursts (generated by CiteSpace).

**Figure 9:**
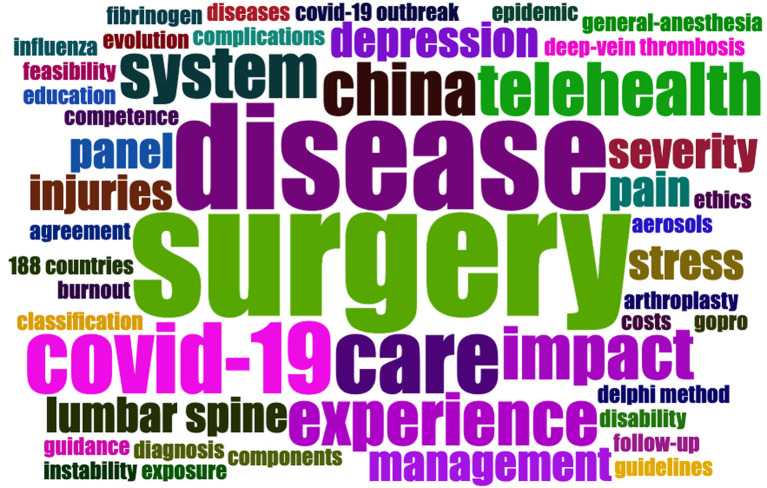
Word cloud. The 50 most frequently occurring keywords are displayed. The keywords are located in the center, and the larger the volume, the more frequently it occurs (generated by Bibliometrix).

Keyword lists are useful for identifying research hotspots and assisting with research. Figure 8 depicts the top 10 keywords with the strongest citation bursts, representing the emphasis of the research field: “elective surgery,” “China,” “elective spine procedure,” “follow up,” “intensive care,” “surgery,” “impact,” “telehealth,” “patient satisfaction,” and “questionnaire.” Figure 9 presents a Word Cloud showing the 50 most frequently occurring keywords. These keywords are located in the center; the larger the volume, the more frequently the keyword occurs.

**Figure 10:**
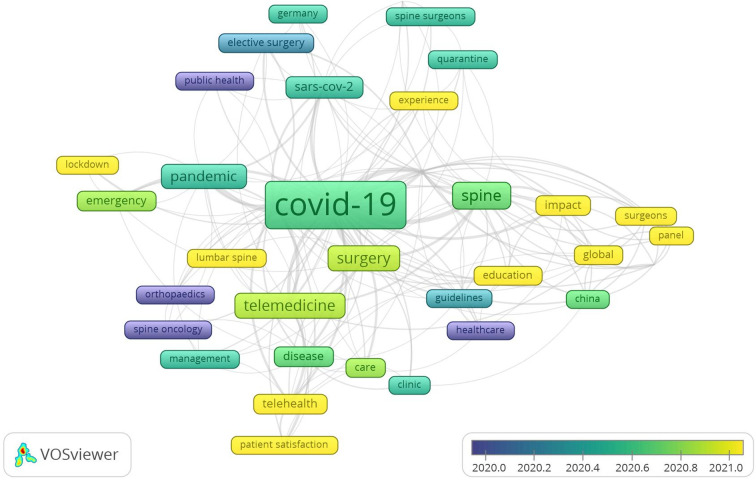
Network diagram of keyword hotspot trends.

In addition, Figure 10 shows the trending network graph for keywords, with a more distant-to-recent time represented as purple-to-yellow. The larger the frames, the more frequently it appears as a keyword, and the distance between the two circles indicates their relevance. Trends in research hotspots in the field of spine surgery and COVID-19 have gradually focused from “early public health” and “healthcare” to more recent “patient satisfaction,” “telehealth,” as well as “experience and education.”

## Discussion

Since the beginning of the COVID-19 pandemic, all departments throughout the healthcare system have been actively involved in combating the disease. During this period, many patients, with suspected or confirmed SARS-CoV-2 infection, asymptomatic infection, or non-infected, requiring spinal surgical treatment presented to the hospitals (15–17). The medical staff throughout the world has been duty-bound to prepare adequately for providing good protection and care, both during and after the surgery, to these patients irrespective of their disease status.

Furthermore, numerous difficulties have arisen in medical practice, including in spine surgery. Various medical organizations across the globe have issued guidelines and recommendations for the provision of competent medical care during the pandemic. However, striking a balance between the task of pandemic preparedness and the need for routine surgery while maintaining regular control of the epidemic has been an issue of grave concern for all surgeons. In this regard, triage is essential for patients who require emergency or urgent surgery, as opposed to those who have the option of elective surgery (18).

Scientific papers provide useful and important information on new medical discoveries and treatment recommendations. Analyzing these publications allows the scholars to learn about the research hotspots and trends and quantitatively assess structural information in their field of study (19). During the COVID-19 pandemic, a lot of literature concerning COVID-19 and spine surgery has been published by researchers around the world in a very short time (9, 20–23). The present study is the first bibliometric analysis in the combined fields of spine surgery and COVID-19, wherein we have comprehensively analyzed the literature, quantified the indicators, and extracted useful information, as well as offered important implications for future studies.

We found that in terms of the number of publications, the United States, China, Egypt, and Argentina contributed the most to research in this field. COVID-19 was first identified and reported in China (24), whereas the United States has had the highest number of COVID-19 infections and deaths (25). The number of COVID-19 cases in the United States increased exponentially, starting in March 2020 (1), which put a tremendous strain on their healthcare system. Likewise, the tenuous state of public health services, inherent deficiencies of the Egyptian economy, and the variable nature of coronavirus influenced the effectiveness of the Egyptian government’s pandemic management capacity, leading to recurrent outbreaks and a sharp increase in the number of patients and deaths (26). Argentina was one of the worst impacted regions in Latin America, with the second greatest number of confirmed cases and the fourth highest mortality rate in Latin America (27). As a result of the exorbitant number of infections and deaths, all these countries have dominated the field of COVID-19 research (including spine surgery) and produced the most influential publications. We also found that regarding international research, the United States had frequent inter-region cooperation, as did various European countries.

A majority of the most cited publications originated in the United States. Notably, none of these papers was published in China, although China was the region of origin of the pandemic. In addition, most of the top 10 articles focused on perioperative response and experience, which highlight the concerns and research hotspots of spine surgeons during COVID-19. Eight of the top 10 institutions involved in the maximum publications belonged to the United States; also, 60% of the top 10 most cited papers were from the United States. Thus, it can be inferred that the United States has made the greatest contribution in the field of research regarding COVID-19 and spine surgery. The most active institutions in this field were the Hospital for Special Surgery and Johns Hopkins University.

Notably, the AOSpine International community contributed 7 articles with 24 citations. Many of the most active authors detected in our analysis are active members of AOSpine and have co-authored many articles. The AOSpine International specializes in research, development, clinical data collection, and education in spine surgery. AOSpine promotes spine surgery worldwide through its commitment to spine surgery research and educational opportunities.

We also observed that the *World Neurosurgery* journal had the largest number of articles published and cited in the combined field of spine surgery and COVID-19. Four of the top 10 most cited papers were published in *World Neurosurgery.* Other journals with notable contributions were the *Global Spine Journal, European Spine Journal, Spine, and Clinical Spine Surgery*. A majority of these journals represent the core journals in the field of spinal surgery and COVID-19 and should be monitored to follow the relevant research trends.

The most frequently cited keywords that helped raise awareness regarding COVID-19 research were “elective surgery,” “intensive care,” “telehealth,” “patient satisfaction,” and “follow-up.” The recent pandemic contributed a great deal to raising awareness about the use of telehealth as a treatment modality in cases where physical interaction with the doctor could be avoided. However, owing to the great demands of the field, spine surgeons have been more concerned about the timing of surgery, perioperative care, treatment standards, and quality of life of the patients undergoing spine surgery. Additionally, the social isolation and strict lockdowns implemented to curb the disease spread resulted in delays in the detection and treatment of spinal disorders (28, 29).

In this study, we also examined the research hotspot trends using VOSviewer’s keyword co-occurrence network and found that during the outbreak, research hotspots in the field of spine surgery gradually shifted from “early healthcare” to “viral management,” and more recently to “experience and education.” Based on our experience and review of the relevant literature, we found the following most important areas regarding spine surgery during the pandemic—surgical timing, preoperative preparation and setup, intraoperative considerations, and postoperative care and follow-up.

### Differentiate between emergency and elective surgery

The scale and severity of the COVID-19 outbreak are unprecedented; therefore, the existing recommendations for spinal surgical practice during the COVID-19 epidemic are limited. There is no consensus regarding the distinction between individuals with a spinal disease who require acute or urgent surgery and those who can wait many months (30, 31). As a principle, surgical intervention for acute nerve compression, spinal cord injury, unstable spinal fractures, and progressive or severe neurological deficits from any cause should not be postponed. Spine surgeons are expected to strictly enforce these triage systems for necessary or elective surgical conditions. They must also follow the local health policy guidelines. For patients who have been identified for surgical treatment, safety management strategies should be strictly followed throughout the surgical process leading to postoperative recovery.

### Preoperative preparation and setup

The operating surgeons must ensure that necessary preoperative examinations for suspected or confirmed patients and asymptomatic infected patients are done, and different types of patients should be examined in batches. During the preoperative examination, specialized personnel should be assigned to accompany the patients, and the designated transfer route should be cleared in advance to reduce the exposure of unrelated personnel. In addition, rational assessment based on the patient’s signs and symptoms, laboratory findings, and radiological data must be involved while selecting the appropriate surgical and anesthesia modalities and explaining the possible risks to the patient and their families.

Patients with suspected or confirmed COVID-19 should be handled in dedicated operating rooms, which may preferably be converted to negative-pressure operating rooms to prevent leakage of contaminated air and protect the external environment from infection (32, 33). In addition, anesthesia, surgical drugs, and instruments must be prepared before the surgery; the involved personnel must be equipped with protective wear equipment and sterilization facilities, and all unrelated articles must be removed (10, 34, 35). The number of participants in the surgery is planned and any surgical personnel is not allowed to enter and leave the operating room at will after entering the operating room; all field supplies are handled by nurses outside the operating room. Throughout this phase, disposable medical consumables and examination instruments were selected, and intraoperative equipment, such as electric knife, ultrasonic knife, and bipolar electrocoagulation, was adjusted to the lowest effective power possible. Two sets of negative-pressure suction devices were prepared—one was placed on the patient’s head and face to reduce the diffusion of respiratory secretions in the air and the other for intraoperative suction of gas fluid (36).

### Intraoperative precautions

Surgeons, anesthesiologists, and nursing staff must be dressed according to the required three levels of protection—personnel on the stage wear disposable protective gowns, disposable surgical gowns, protective slippers and shoe covers, medical protective masks, goggles or protective face screens, and at least double gloves, in addition to hand-washing clothes (9, 10). The surgeons should promptly attract smoke generated during surgery to avoid aerosol damage, avoid occupational exposure, and alert the surrounding personnel in advance when splashes of blood, body fluids, or bone chips are caused by the use of knives, drills, and bone cutters; use disposable waterproof materials to cover them up. The anesthesiologists have been using a visual laryngoscope with a disposable laryngeal lens, and the monitor and handle are protected with a protective sleeve. During the preoxygenation phase of anesthesia induction, two wet gauzes are recommended to cover the patient’s mouth and nose before mask ventilation. Muscarinic medication is used in sufficient doses at once to avoid choking and coughing when the patient is intubated. Using an artificial nose intraoperatively is recommended to effectively prevent contamination of the anesthesia machine from bacteria and viruses, and care should be taken to change it every 3 to 4 h.

### Postoperative precautions

Patients with COVID-19 should be transported using special access and elevators. After surgery, the patient must be returned to the original isolation ward with monitoring equipment or negative-pressure isolation care unit, and actively treated with analgesia, anti-infection, rehydration, and multidisciplinary combination therapy, if necessary. Regular repeat COVID-19 testing is certainly required.

### Telemedicine

To ensure the safety of patients and clinicians while ensuring competent clinic care, spine clinics must adapt to the post-COVID-19 era. Concurrently, patient access and care practices have changed due to greater hygiene awareness, the use of advanced personal protective equipment, and more digital communication options. Telemedicine allows the clinician and the patient to diagnose spinal disorders if both have the capacity and infrastructure to do so. During this pandemic, telemedicine has helped surgeons reduce their exposure risk while allowing patients to remain at home and follow public health recommendations (37). It also allows for a postoperative follow-up to provide immediate feedback on postoperative recovery or complications.

### Limitations

We performed an objective and thorough review of the data in the publications on spine surgery and COVID-19 in the WoS database. Nevertheless, some limitations were unavoidable in this study. First, literature from only one database was included in this study, therefore, some meaningful and relevant papers may have been missed. Second, although many new research articles are added to the WoS daily, a portion is not included in the Core Collection. Third, certain recently published high-quality articles had a smaller number of citations due to the short interval between the time of publication and this study, which may have impacted the total citation frequency of our research. Fourth, the results may vary depending on the search time, more so regarding the number of citations. Finally, because of the specificity of our study topic (spine surgery and COVID-19), only limited literature could be included for analysis.

## Conclusions

With the advance of the pandemic, an increasing number of research articles are being published in the field of spine surgery and COVID-19; however, it is important to assess the quality of this extensive literature to extract meaningful information. During this pandemic, spine surgeons have been more concerned with the surgical timing, care, treatment, and the patient’s quality of life. Accordingly, the research hotspots and trends in this field changed from early public health to virus and management and more recently to surgery-related experience and education.

## Data Availability

The original contributions presented in the study are included in the article/Supplementary Material, further inquiries can be directed to the corresponding author/s.
